# Obesity in Tibetans Aged 30–70 Living at Different Altitudes under the North and South Faces of Mt. Everest

**DOI:** 10.3390/ijerph7041670

**Published:** 2010-04-13

**Authors:** Lhamo Y. Sherpa, Deji, Hein Stigum, Virasakdi Chongsuvivatwong, Dag S. Thelle, Espen Bjertness

**Affiliations:** 1 Section for Preventive Medicine and Epidemiology, University of Oslo, Frederik Holsts hus, Ulleval terrasse, Kirkeveien 166, 0450 Oslo, Norway; E-Mail: Hein.Stigum@fhi.no (H.S.); espen.bjertness@medisin.uio.no (E.B.); 2 Tibet University Medical College, No.1 Loubulinka Road, Lhasa, Xizang 850002, China; E-Mail: dejioslo@yahoo.com; 3 National Institute of Public Health, Oslo, Norway; 4 Epidemiology Unit, Prince of Songkla University, Hat Yai, Songkhla 90110, Thailand; E-Mail: cvirasak@medicine.psu.ac.th; 5 Institute of Basic Medical Sciences, University of Oslo, Oslo, Norway; E-Mail: d.s.thelle@medisin.uio.no; 6 Nepal Institute of Health Sciences affiliated to Purbanchal University, Kathmandu, Nepal

**Keywords:** obesity, WHtR, BMI, waist circumference, Tibetans, Tibet, Everest, Nepal

## Abstract

Risk factors for chronic diseases in Tibetans may be modified due to hypobaric hypoxia. The objectives of this study were to determine the prevalence of obesity at varying altitudes of 1,200, 2,900 and 3,700 meters above sea-level in Tibet and Nepal; to estimate the effect of altitude on body mass index (BMI), waist circumference (WC) and waist-to-height ratio (WHtR). Three cross-sectional studies with simple random sampling were performed on 617 men and women. BMI, WC and WHtR decreased with increasing altitude. It is likely that the physical conditions such as low temperatures and low oxygen levels have a direct catabolic effect.

## 1. Introduction

Obesity is a result of higher energy intake than expenditure, but the mechanisms are complex and not well understood [1]. The prevalence of obesity disproportionately affects certain racial/ethnic populations, with a higher rate among certain ethnic minority groups [2] ethnic admixture adults [3], aboriginal populations [4] and high altitude natives [5,6]. Factors that predict obesity among different ethnic groups may vary owing to environmental differences like childhood undernutrition and overnutrition during adulthood [7], sedentary lifestyle [8] and limited access to education and healthier foods [2,8]. Altitude is an environmental factor which may be associated with obesity [9]. Studies among sojourns and lowlanders acclimatized to high altitudes have reported appetite suppression and weight loss under hypoxic conditions [10–12]. However, these findings are inconsistent due to differences in thermoregulatory response to cold environments between people with high and low basal metabolic rates [9,13]. Santos *et al*. [14] reported a prevalence of 23.5% of high body mass index (BMI ≥ 30 kg/m^2^) among 196 Aymara natives living above 2,000 meters of altitude, indicating that obesity is also a public health problem in poor and high altitude populations. Of the World population about 17 million live at altitudes higher that 3,500 meters and more than 140 million live above 2,500 meters [15]. As far as the authors are aware of, there are no published studies linking altitude to obesity in a well defined high altitude population.

Tibetans belong to the world’s oldest mountainous community residing at varying altitudes, mainly above 3,500 meters. Tibetans in Nepal migrated from the Eastern part of Tibet approximately 450 years ago. Tibetans in both countries still share the same culture and traditional foods and are exposed to different severity of hypoxia depending on the altitude of residence. They share the same genes helping life at high altitude.

The aims of the present study were to estimate the prevalence of obesity among 30–70 year old male and female Tibetans living at varying altitudes of 1,200, 2,900 and 3,600 meters above sea-level in Nepal and Tibet, and to investigate the effect of altitude in body mass index (BMI), waist circumference (WC) and waist-to-height ratio (WHtR).

## 2. Population and Methods

### 2.1. Study Population and Methods

Three cross-sectional studies were conducted in Lhasa (Tibet) at 3,660 m and at 1,200 and 2,900 meters in the Everest region of Nepal. The data collections were done in Tibet in 2006 (September–December) and 2007 (May–August) in Nepal, respectively, with corresponding temperature ranges of: 22 °C–33 °C at 1,200 meters, 10 °C–15 °C at 2,900 meters, and between 7 °C and 10 °C at 3,660 meters, respectively.

In Tibet, out of 28 resident committees, four were randomly selected. Five hundred thirty seven randomly selected individuals aged 30–70, were invited to participate. Three hundred seventy one (69%) participated in the survey.

In Nepal, at 2,900 and 1,200 meters of altitude, 120 (88%) and 126 (92%) joined the study out of 136 invitees at each site. Unwillingness to undergo venipuncture was the main reason for not participating. More details about the samples from Nepal have been published elsewhere [16].

The interview questionnaires were adapted from the WHO MONICA project. Some of the questions were modified according to local conditions. A simple clinical examination was also performed. Data collectors included teams of trained teachers, nurses and laboratory personnel. Approval for the study was received from the Norwegian Regional Committees for Medical and Health Research Ethics (REK) (Lhasa study), Tibet Autonomous region (TAR) (Lhasa study) and National Health Research Council of Nepal (Nepal studies). Permission was also granted by the selected counties and villages to conduct studies. All the participants gave written and oral consent after being explained the procedure of the study, and were allowed to withdraw from the study sample at any time without any consequences.

## 2.2. Variables

Body height and weight were measured with the participant in light clothing without shoes. Height was measured to the nearest centimeter with a vertical ruler and weight to the nearest kilogram with a spring balance. Calibration was done after every 10th measurement both in Nepal and Tibet. Body mass index (BMI) was calculated as the weight in kilograms divided by the height in meters squared. Waist circumference (WC) was measured by encircling the tape measure at the midpoint between the lower costal margin and the superior iliac crest after exhaling. Following the WHO protocol [17] for BMI, obesity was defined at a BMI of ≥30. Using the Adult Treatment Panel III criteria [18], participants were also divided into two categories: abdominally obese (WC > 88 cm for women and WC > 102 cm for men) or non-abdominally obese. Waist-to-height ratio (WHtR) was calculated by dividing waist circumference by height. Altitude was operationalized based on the three populations groups (samples). We constructed a continuous variable measuring altitude in units of 1,000 meters, giving three values for altitude: 1.2, 2.9 and 3.66.

Using the International Physical Activity Questionnaire (IPAQ), metabolic equivalent of tasks (MET) scores were calculated for physical activity for data from Nepal. A dietary questionnaire was developed with the assistance of an experienced nutritionist for data from Nepal. Caloric intake was assessed by calculating the most frequent foods taken during breakfast, lunch and dinner. Calories of individual food items were calculated and pictures of portion sizes were shown to the respondent.

Monthly income and current job situation of either having (“Yes”) or not having a job (“No”) was assessed. Education was also categorized into “Yes” and “No” depending on their school level attendance. Alcohol consumption was classified as either “Yes” if the person drank alcohol regularly (more than five days a week), otherwise “No”. Smoking was classified as either “regular” if the person smoked daily, “occasional” if the person smoked a few times a week or “No” if the person had stopped smoking during the past 12 months. An average number of cigarettes per day was also sought.

### 2.3. Statistical Analysis

**Figure 1.**
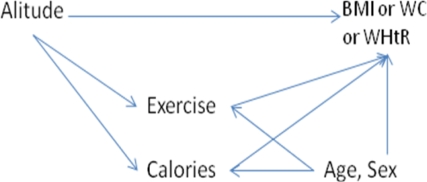
Diagram of possible pathways.

Characteristics and obesity parameters of the subjects by altitude of residence were described in medians and frequencies. The distribution of the outcome variables BMI, waist circumference and waist-to-height ratio were skewed, with long tails to the right. Therefore non-parametric (Kruskal-Wallis) test were used when comparing the BMI, WC or WHtR distributions between groups. Chi-squared method was used for comparing proportion of obese people. Using data from Nepal, multiple linear regressions were used to find the direct effect of altitude on BMI, WC and WHtR individually, and the indirect effect passing through exercise and calorie intake (Figure 1). The effects of exercise and calories were adjusted for age and sex. Because of the skewness of the outcome variables, the linear models were carefully tested for deviations from linearity, and for non-constant residual variation (heteroscedasticity). The robustness of the models against outliers were tested by plotting delta-beta values. A p-value of ≤0.05 was considered statistically significant. Data were analyzed using R 2.9.2 [19].

## 3. Results and Discussion

### 3.1. Results

Characteristics of subjects by altitude of residence are described in Table 1. Participants at 1,200 meters were older than those at the other two altitudes. A higher number of participants at 3,660 meters were smokers and were educated. The proportion of people with jobs was higher among participants at 2,990 meters of altitude, with the lowest average income.

Table 2 illustrates obesity parameters by altitude of residence. BMI, WC, WHtR and obesity decreased with increasing level of altitude.

**Figure 2.**
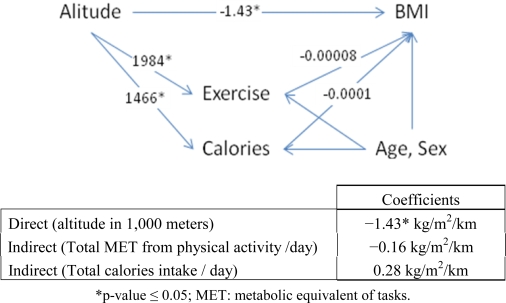
Path diagram for the effect of altitude on body mass index (BMI).

**Figure 3.**
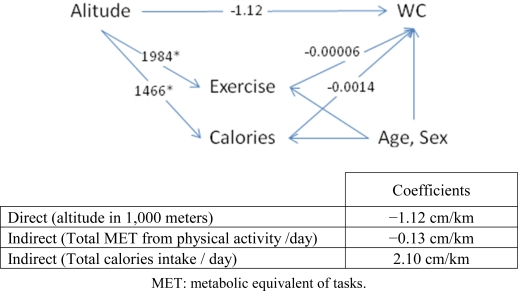
Path diagram for the effect of altitude on waist circumference (WC).

**Figure 4.**
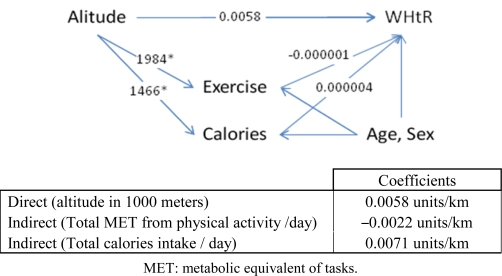
Pathdiagram for the effect of altitude on waist-to-height ratio (WHtR).

Figures 2, 3 and 4 describe the effects of altitude on obesity parameters using the Nepal data. Figure 2 describes the effects of altitude on BMI. The direct effect of altitude on BMI was −1.43 kg/m^2^/km after adjusting for other variables in the model. Physical activity and calorie consumption at higher altitudes increased significantly by 1,984 metabolic equivalent tasks (METs) and 1,466 kilocalories (kcal) per altitude, respectively. The effect of physical activity and calories on BMI after controlling for age and sex was −0.00008 kg/m^2^/MET and 0.0001 kg/m^2^/kcal, respectively. The indirect effects of altitude on BMI when passed through intermediate variables physical activity and calories were −0.16 kg/m^2^/km, and 0.28 kg/m^2^/km, respectively, after adjusting for age and sex. The reduction in BMI explained by physical activity and calories was minimal. The total effect of altitude on BMI then equaled −1.43 − 0.16 + 0.28 = −1.31 kg/m^2^ per kilometer of altitude.

Figure 3 illustrates the effect of altitude on waist circumference. The direct effect of altitude on WC was −1.12 cm/km after adjusting for other variables in the model. Physical activity and calorie consumption increased significantly by 1,984 METs and 1,466 kcal per altitude, respectively. The effect of physical activity and calories on WC after controlling for age and sex was −0.00006 cm/MET and 0.0014 cm/kcal, respectively. The indirect effects of altitude on WC when passed through the intermediate variables physical activity and calories were −0.13 cm/km, and 2.10 cm/km, respectively, when also adjusted for age and sex. Physical activity decreased WC by 0.13 cm/km, similarly calorie intake increased WC by 2 cm/km. The total effect of altitude on WC then equaled −1.12 − 0.13 + 2.10 = 0.85 cm per kilometer of altitude.

## 3.2. Discussion

The main findings of the present study are that the prevalence of obesity and central obesity are common, with a significant decrease with an increasing level of altitude. Calorie intake and physical activity could not explain the lower prevalence of BMI, WC and WHtR. However, the combined effect of altitude on BMI, WC and WHtR were different, as high altitude significantly reduced BMI, but not WC and WHtR. Increase in calorie intake at higher altitudes was associated with an increase in waist circumference but not BMI. The effect on waist circumference was reduced when adjusted for height. Many studies done among sojourns with small sample sizes have reported weight loss under hypoxic conditions, particularly due to a decrease in food intake [11,12]. In contrast, our study found increased calorie consumption at higher altitudes. A reason for increase in calories intake could be due to lower basal metabolic rate among high altitude natives [9,20]. People with low basal metabolic rate have weak peripheral vasoconstriction responses to cold. The core temperature of these subjects is influenced by cold and the ability to suppress heat loss is reduced, thereby increasing metabolic heat production through increased calorie consumption. Another reason for lower BMI, WC and WHtR despite increased caloric intake could also be due to elevated physical activity level at higher altitudes, as demonstrated in this study. It has been suggested that differences in energy selecting patterns occur among high altitude natives when synergistically exposed to cold and hypoxia. Some studies have suggested that weight loss at higher altitudes among acclimatized highlanders is due to reduction in body fat due to fat catabolism [9,21,22]. When there is imbalance between energy intake and energy utilized the additional resulting energy deficit is offset by fat catabolism. Other studies have on the other hand suggested that utilization of carbohydrate is more pronounced during shivering mechanism at higher altitudes as this is more oxygen efficient [23–25], but non-shivering mechanisms among higher altitude population have also been documented [9,20]. Similarly, genetic and adaptive differences in oxygen consumption among high altitude natives have been reported [26,27].

There were more smokers among the Tibetans residing at 3,660 meters of altitude. Smokers were less likely to be overweight [28,29], however, other serious health hazards like lung cancer associated with smoking should not be underestimated. No smokers were identified at 2,900 meters of altitude. Frequent non-smoking campaign in this Everest region of Nepal could have influenced people to quit smoking.

More than a quarter of population at all the three sites were alcohol consumers. Alcohol consumption leads to fat deposition in the abdominal region [30,31], hence a higher alcohol consumption rate in our study may also be associated with a higher abdominal obesity.

In the recent years, many studies have suggested loss of weight at high altitudes possibly due to increased level of leptin [32,33]. However, the role of leptin remains controversial. Study done among animals [34] and human beings at high altitude [35–37] have found decreased levels of leptin. Likewise a study comparing sea level residents with high altitude natives found no statistical difference in plasma leptin levels [37]. Plasma leptin tends to increase in hypoxic conditions whereas in cold temperature production of leptin is inhibited. Therefore, the synergistic effect of hypoxia and cold temperature to leptin among high altitude natives still needs to be elucidated.

Energy expenditure was not measured in the present study, and the proportion of energy intake to energy output could therefore not be measured. Neither did we have data on changes in appetite due to cold, stress or a qualitative or quantitative limited food supply. Genetic differences might contribute to the observed differences, although these two groups from Tibet and Nepal share a similar ancestry. It may be possible that some adaptive changes could have occurred among those residing in Nepal over the past few hundred years. Participants from Tibet and Nepal differ in the degree of physical activity and the altitude of residence. Participants in Tibet at 3,660 meters and in Nepal at 1,200 meters have recently gained access to modern amenities whereas those residing at 2,900 meters in Nepal still do not have access to transportation and hospitals. Data collection in Tibet was conducted in autumn and winter, whereas in Nepal it was collected during spring and summer. Since Tibet is at a higher altitude with colder temperature it may be possible that the demand for heat production was greater during data collection period. Climate variation may have biased the results towards lower prevalence of obesity and central obesity in Tibet. Seasonal migration among high altitude natives is quite common owing to wage earning activities. However, since a larger proportion of the population in our study were females who are less mobile, the effect of migration on obesity prevalence should have been minimal. Finally, the limitation of subjective measure of physical activity measuring non-structured form of physical activity may have provided uncertain information about the effect of physical activity on obesity. Further research with objective measures of physical activity and assessments of energy expenditure in high altitude populations is needed.

## 4. Conclusion

BMI, WC and WHtR decreased with increasing altitude. The mechanism for these differences are not known, and we were not able to explain this by lower energy intake or increased physical activity. It is likely that the physical conditions such as low temperatures and low oxygen levels have a direct catabolic effect.

## Figures and Tables

**Table 1.** Characteristics of subjects by altitude of residence.

	1,200 meters (n = 127)	2,900 meters (n = 119)	3,660 meters (n = 371)
Age			
Median (IQR)	50 (41–59)	42 (35–54)	46 (39–57)
Gender			
Male	35 (27.6)	35 (29.4)	139 (37.5)
Female	92 (72.4)	84 (70.6)	232 (62.5)
Alcohol > 5times / week			
Yes	44 (34.9)	67 (56.3)	164 (44.4)
No	82 (65.1)	52 (43.7)	205 (55.6)
Current smoking			
Regularly	2 (1.6)	0 (0)	88 (23.7)
Occasionally	1 (0.8)	0 (0)	2 (0.5)
Never	24 (97.6)	119 (100)	281 (75.7)
average no. of cig. Median (IQR)	4 (3.5–5.5)	0 (0)	20 (10–20)
Education			
Yes	48 (37.8)	33 (27.7)	217 (59)
No	79 (62.2)	86 (72.3)	51 (41)
Having a job			
Yes	27 (21.3)	116 (97.5)	206 (55.7)
No	100 (78.7)	3 (2.5)	164 (44.3)
Income/month (in Yuan)			
50–900	10 (7.9)	80 (67.2)	210 (56.6)
901–2000	10 (7.9)	26 (21.8)	52 (14)
>2000	7 (5.5)	8 (6.7)	28 (7.5)
None	100 (78.7)	5 (4.2)	81 (21.8)

**Table 2.** Obesity parameters by altitude of residence.

Median (IQR)	1,200 meters	2,900 meters	3,660 meters	p-value
BMI^a^(kg/ m^2^)	26.2 (23.9–28.6)	24.5 (21.8–27.6)	24.6 (22.1–27.2)	<0.001
WC^b^ (cms)	94.0 (85–101.2)	92.0 (83–101)	84.5 (76.5–92)	<0.001
WHtR^c^	0.6 (0.5–0.7)	0.6 (0.5–0.6)	0.5 (0.5–0.6)	<0.001
Central Obesity^d^				
Yes	68 (53.5)	68 (57.1)	92 (24.8)	<0.001
No	59 (46.5)	51 (42.9)	279 (75.2)	
Obesity^e^				
Yes	25 (19.7)	14 (11.8)	36 (9.7)	0.012
No	102 (80.3)	105 (88.2)	335 (90.3)	

a body mass index;

b waist circumference;

c waist-to-height ratio;

d WC > 102 for men & WC > 88 cms for women;

e BMI ≥ 30.

## References

[1] Lambert EV Goedecke JH Energy balance and energy expenditure in obesity—Is obesity a disease of inactivity? SA Sports Medicine 2003 15 21 25

[2] Ong SK Fong CW Ma S Lee J Heng D Deurenberg-Yap M Low YL Tan M Lim WY Tai ES Longitudinal study of the socio-demographic determinants of changes in body weight and waist circumference in a multi-ethnic Asian population Int. J. Obes. (Lond) 2009 33 1299 1308 19736555 10.1038/ijo.2009.173

[3] Albright CL Steffen AD Wilkens LR Henderson BE Kolonel LN The prevalence of obesity in ethnic admixture adults Obesity 2008 16 1138 1143 18356848 10.1038/oby.2008.31 PMC4479279

[4] Lindgärde F Ercilla MB Correa LR Ahrén B Body adiposity, insulin, and leptin in subgroups of Peruvian Amerindians High Alt. Med. Biol 2004 5 27 31 15072714 10.1089/152702904322963663

[5] Mohanna S Baracco R Seclén S Lipid profile, waist circumference, and body mass index in a high altitude population High Alt. Med. Biol 2006 7 245 255 16978137 10.1089/ham.2006.7.245

[6] Shah SM Nanan D Rahbar MH Rahim M Nowshad G Assessing obesity and overweight in a high mountain Pakistani population Trop. Med. Int. Health 2004 9 526 532 15078272 10.1111/j.1365-3156.2004.01220.x

[7] McMillen IC Robinson JS Developmental origins of the metabolic syndrome: prediction, plasticity, and programming Physiol. Rev 2005 85 571 633 15788706 10.1152/physrev.00053.2003

[8] Caballero B Introduction. Symposium: Obesity in developing countries: biological and ecological factors J. Nutr 2001 131 866 870 10.1093/jn/131.3.866S 11238776

[9] Davis TR The influence of climate on nutritional requirements Am. J. Public Health Nations Health 1964 54 2051 2067 14240513 10.2105/ajph.54.12.2051 PMC1255122

[10] Guilland JC Klepping J Nutritional alterations at high altitude in man Eur. J. Appl. Physiol. Occup. Physiol 1985 54 517 523 4085482 10.1007/BF00422963

[11] Tschöp M Strasburger CJ Hartmann G Biollaz J Bärtsch P Raised leptin concentrations at high altitude associated with loss of appetite Lancet 1998 352 1119 1120 10.1016/S0140-6736(05)79760-9 9798594

[12] Westerterp-Plantenga MS Westerterp KR Rubbens M Verwegen CR Richelet JP Gardette B Appetite at “high altitude” [Operation Everest III (Comex-’97)]: a simulated ascent of Mount Everest J. Appl. Physiol 1999 87 391 399 10409600 10.1152/jappl.1999.87.1.391

[13] Westerterp-Plantenga MS Effects of extreme environments on food intake in human subjects Proc. Nutr. Soc 1999 58 791 798 10817145 10.1017/s002966519900107x

[14] Santos JL Pérez-Bravo F Carrasco E Calvillán M Albala C Low prevalence of type 2 diabetes despite a high average body mass index in the Aymara natives from Chile Nutrition 2001 17 305 309 11369169 10.1016/s0899-9007(00)00551-7

[15] Hornbein TF Schoene RB High Altitude: An Exploration of Human Adaptation Lung Biology in Health and Disease Lenfant C Marcel Dekker, Inc New York, NY, USA 2001 161 67

[16] Lhamo SY Supamai S Virasakdi C Impaired glucose regulation in a Sherpa indigenous population living in the Everest region of Nepal and in Kathmandu Valley High Alt. Med. Biol 2008 9 217 222 18800958 10.1089/ham.2008.1011

[17] WHO/IASO/IOTF The Asia-Pacific Perspective: Redefining Obesity and Its Treatment Health Communications Australia Melbourne, Australia 2000ISBN 0-9577082-1-1.

[18] Expert Panel on Detection, Evaluation, and Treatment of High Blood Cholesterol in Adults Executive Summary of the Third Report of the National Cholesterol Education Program (NCEP) Expert Panel on Detection, Evaluation, and Treatment of High Blood Cholesterol in Adults (Adult Treatment Panel III) JAMA 2001 285 2486 2497 11368702 10.1001/jama.285.19.2486

[19] R Development Core Team 2009Available online: http://www.r-project.org/ (accessed on 7 October 2009).

[20] Maeda T Fukushima T Ishibashi K Higuchi S Involvement of basal metabolic rate in determination of type of cold tolerance J. Physiol. Anthropol 2007 26 415 418 17641462 10.2114/jpa2.26.415

[21] Guilland JC Klepping J Nutritional alterations at high altitude in man Eur. J. Appl. Physiol. Occup. Physiol 1985 54 517 523 4085482 10.1007/BF00422963

[22] Boyer SJ Blume FD Weight loss and changes in body composition at high altitude J. Appl. Physiol 1984 57 1580 1585 6520055 10.1152/jappl.1984.57.5.1580

[23] Hochachka PW Exercise limitations at high altitude: the metabolic problem and search for its solution Circulation, Respiration and Metabolism Gilles R Berlin and Heidelberg Springer-Verlag, New York, NY, USA 1985 240 249

[24] Haman F Péronnet F Kenny GP Massicotte D Lavoie C Weber JM Partitioning oxidative fuels during cold exposure in humans: Muscle glycogen becomes dominant as shivering intensifies J. Physiol 2005 566 247 256 15831534 10.1113/jphysiol.2005.086272 PMC1464733

[25] Brooks GA Butterfield GE Wolfe RR Groves BM Mazzeo RS Sutton JR Wolfel EE Reeves JT Increased dependence on blood glucose after acclimatization to 4,300 m J. Appl. Physiol 1991 70 919 927 2022585 10.1152/jappl.1991.70.2.919

[26] Beall CM High-altitude adaptations Lancet 2003 362 14 15 14698112 10.1016/s0140-6736(03)15058-1

[27] Brutsaert TD Do high-altitude natives have enhanced exercise performance at altitude? Appl. Physiol. Nutr. Metab 2008 33 582 592 18461115 10.1139/H08-009

[28] Albanes D Jones DY Micozzi MS Mattson ME Associations between smoking and body weight in the US population: analysis of NHANES II Am J Public Health 1987 77 439 444 3493709 10.2105/ajph.77.4.439 PMC1646954

[29] Fang H Ali MM Rizzo JA Does smoking affect body weight and obesity in China? Econ. Hum. Biol 2009 7 334 350 19660996 10.1016/j.ehb.2009.07.003

[30] Kvist H Hallgren P Jönsson L Pettersson P Sjöberg C Sjöström L Björntorp P Distribution of adipose tissue and muscle mass in alcoholic men Metabolism 1993 42 569 573 8492711 10.1016/0026-0495(93)90214-9

[31] Suter PM Is alcohol consumption a risk factor for weight gain and obesity? Crit. Rev. Clin. Lab. Sci 2005 42 197 227 16047538 10.1080/10408360590913542

[32] Shukla V Singh SN Vats P Singh VK Singh SB Banerjee PK Ghrelin and leptin levels of sojourners and acclimatized lowlanders at high altitude Nutr. Neurosci 2005 8 161 165 16117183 10.1080/10284150500132823

[33] Tschöp M Strasburger CJ Töpfer M Hautmann H Riepl R Fischer R Hartmann G Morrison K Appenzeller M Hildebrandt W Biollaz J Bärtsch P Influence of hypobaric hypoxia on leptin levels in men Int. J. Obes. Relat. Metab. Disord 2000 24 151 10997640 10.1038/sj.ijo.0801309

[34] Norese MF Lezon CE Alippi RM Martinez MP Conti MI Bozzini CE Failure of polycythemia-induced increase in arterial oxygen content to suppress the anorexic effect of simulated high altitude in the adult rat High Alt. Med. Biol 2002 3 49 57 12006164 10.1089/152702902753639540

[35] Santos JL Pérez-Bravo F Albala C Calvillán M Carrasco E Plasma leptin and insulin levels in Aymara natives from Chile Ann. Hum. Biol 2000 27 271 279 10834292 10.1080/030144600282163

[36] Vats P Singh SN Shyam R Singh VK Singh SB Banerjee PK Selvamurthy W Leptin may not be responsible for high altitude anorexia High Alt. Med. Biol 2004 5 90 92 15072723 10.1089/152702904322963753

[37] Woolcott OO Castillo OA Torres J Damas L Florentini E Serum leptin levels in dwellers from high altitude lands High Alt. Med. Biol 2002 3 245 246 12162867 10.1089/15270290260131975

